# Roles of divalent metals in the regulation of cyclic di-GMP signaling in bacteria

**DOI:** 10.1128/jb.00605-25

**Published:** 2026-07-10

**Authors:** Aathmaja Anandhi Rangarajan, Christopher M. Waters

**Affiliations:** 1 Department of Microbiology, Genetics and Immunology, Michigan State University 220155, East Lansing, Michigan, USA; Dartmouth College Geisel School of Medicine, Hanover, New Hampshire, USA

**Keywords:** calcium, iron, manganese, magnesium, zinc, cyclic di-GMP, metal

## Abstract

Metals are essential co-factors that are important for the activity of many enzymes and, hence, bacterial life itself. Cyclic di-GMP (c-di-GMP) is an important signaling molecule that is prevalent across various bacterial phyla and involved in myriad physiological processes. The structure and function of diguanylate cyclase (DGCs) and phosphodiesterase (PDEs) domains that make and degrade c-di-GMP are also highly conserved. These enzymes are influenced by several signaling cues, such as temperature, oxygen, and small molecules, which determine the intracellular c-di-GMP levels and subsequent bacterial physiology. In this review, we summarize the current knowledge of the roles of divalent metals Mn^2+^, Mg^2+^, Zn^2+^, Ca^2+^, and Fe^2+^ in modulating the activity of DGCs and PDEs and c-di-GMP-related phenotypes. We describe the role of divalent metals in modulating DGC and PDE catalysis, and then discuss the examples of divalent metals as signals that modulate c-di-GMP levels. We also discuss how metals can influence the transcription of c-di-GMP catalytic enzymes. The review highlights the underexplored question of how metal availability shapes c-di-GMP signaling across diverse environmental contexts.

## INTRODUCTION

**Fig 1:**
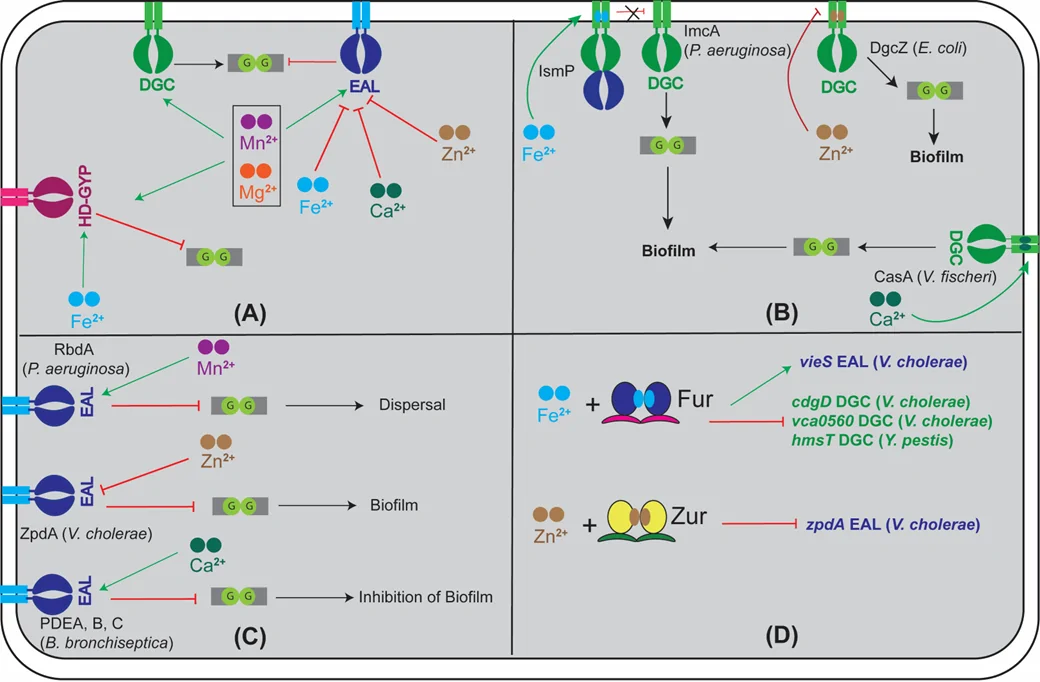
Metals affecting various aspects of c-di-GMP signaling in bacteria. (**A**) Metals affecting catalytic domain of c-di-GMP dependent enzymes. (**B**) Metals affecting signaling domain of c-di-GMP-dependent enzymes. (**C**) Metals affecting c-di-GMP enzymes via unknown mechanism. (**D**) Metals affecting c-di-GMP gene expression via metalloregulatory transcription factors.

Bacteria thrive in a wide range of environments, from soil and water to colonization of host organisms, and they must rapidly adapt to fluctuating conditions to survive and propagate. One of the key mechanisms underlying this adaptability is the use of nucleotide-based secondary messengers, which detect environmental stressors, amplify intracellular signals, and trigger changes in transcriptional, translational, and post-translational programs. Among these messengers, c-di-GMP is an ubiquitous signaling molecule found in the majority of bacterial phyla (1, 2). Primarily recognized for its role in inducing biofilm formation and inhibiting motility, c-di-GMP also orchestrates a broad spectrum of physiological processes, including DNA repair, secretion systems, cell shape, animal and plant colonization, cell cycle progression, aggregation, chemotaxis, antibiotic persistence, phage resistance, and virulence, etc (3–10). The intracellular concentration of c-di-GMP is tightly controlled by opposing activities of diguanylate cyclases (DGCs), which synthesize c-di-GMP from GTP, and phosphodiesterases (PDEs), which degrade c-di-GMP into pGpG or GMP (Fig. 1). DGC enzymes contain a GGDEF domain, while PDE enzymes contain an EAL or HD-GYP domain. Both DGCs and PDEs are often equipped with N-terminal sensory domains that respond to specific environmental cues (1). While a handful of direct signals, such as nitric oxide, bile, spermidine, light, oxygen, temperature, nucleotides, amino acids, and quorum sensing molecules, have been identified, the full spectrum of stimuli modulating c-di-GMP metabolism remains incompletely understood (11, 12).

One critical yet underexplored category of environmental signals that modulates c-di-GMP signaling is divalent metals. Bacteria frequently encounter fluctuating metal concentrations as they colonize diverse niches, including human hosts, plants, aquatic systems, and soil (13–15). Metal co-factors, such as Zn^2+^, Fe^2+^, Mn^2+^, Mg,^2+^ and Co^2+^, are essential for the activity of over a third of bacterial proteins (16, 17). Since living organisms cannot synthesize metals, bacteria must import them from the environment into the cell. Hence, these metal co-factors exist in a narrow concentration inside the bacteria, and their import and export are tightly regulated in order to maintain homeostatic levels, failure of which leads to cell death (18–20). Like most enzymes, c-di-GMP metabolizing DGCs and PDEs need metals as co-factors for their activity. The connection between metal ions and c-di-GMP signaling was first observed during the discovery of c-di-GMP itself, where Moshe Benziman and colleagues showed PDEs that degrade c-di-GMP in *Komagataeibacter xylinus* (formerly *Acetobacter xylinum*) were inhibited by Ca^2+^ ions, which increased cellulose synthesis (21, 22). Following this observation, many proteins possessing GGDEF, EAL, and HD-GYP enzymes have been isolated, crystallized, and tested for their activity to make or degrade c-di-GMP, and all of them require metal cofactors for their catalytic activity. Emerging research continues to reveal how divalent metals modulate c-di-GMP levels and influence associated bacterial phenotypes.

In this review, we summarize the current literature on the diverse roles of divalent metals in regulating c-di-GMP metabolism. We focus on the effects of Mn^2+^, Mg^2+^, Zn^2+^, Fe^2+^, and Ca^2+^ and their effect on c-di-GMP levels by their impact on DGCs and PDEs both catalytically and as environmental signals.

## IMPACT OF DIVALENT METALS ON THE CATALYTIC DOMAIN OF C-DI-GMP MODULATING ENZYMES

Metals can directly impact DGCs and PDEs either through modulating catalysis at the enzyme active site or by functioning as a signal by binding to the N-terminal signaling domain. We will first discuss the literature exploring the diverse impact of metals on the catalytic activity of c-di-GMP-associated enzymes via their GGDEF, EAL, or HD-GYP catalytic domains.

## MG^2+^ AND MN^2+^ DRIVE ENZYMATIC ACTIVITYOF GGDEF- AND EAL-CONTAINING PROTEINS

GGDEF domain-containing DGCs are ubiquitous across the bacterial kingdom (2). Dimerization is crucial for c-di-GMP synthesis, as each GGDEF monomer binds one GTP molecule. The two GTPs align in an anti-parallel orientation, enabling a nucleophilic attack between their α-phosphates, resulting in c-di-GMP formation and pyrophosphate release (23, 24). Mg²⁺ plays a central role in this reaction by coordinating aspartate or glutamate in the conserved GG(D/E)EF active site with the β- and the γ-phosphates of GTP and the first aspartate in the DxD motif on the β1 strand (24, 25). Crystal structures of GGDEFs often reveal two Mg²^+^ ions bound at these sites, and mutational analyses of these metal-binding residues confirm their essential role in catalytic activity by enabling dimerization and stabilizing the divalent state during cyclization (26–29). It is important to note that most structural studies rely on crystallization conditions rich in Mg²⁺, potentially overlooking the roles of other biologically relevant metals in catalysis. In addition to Mg²⁺, Mn²⁺ also supports enzymatic activity of many GGDEF-containing proteins (Fig. 1A) (Table 1) (25, 26, 30). A well-characterized GGDEF-containing protein, PleD, exhibits enhanced DGC activity with both Mg²⁺ and Mn²⁺, with Mn²⁺ promoting stronger dimerization (25). Hence, while Mg²⁺ promotes DGC catalytic activity and is bound in many crystal structures of GGDEFs, Mn^2+^ could displace Mg^2+^ ions under high Mn^2+^ conditions in GGDEF-containing proteins, resulting in enhanced dimerization and activity.

**TABLE 1:** Metal ion regulation of GGDEF domain containing DGCs^*a*^

Metals regulating GGDEF domain containing DGCs	Protein	Organism	Crystal structure with Mg^2+^	*In vitro* protein activity tested	Reference
Mg^2+^(+), Mn^2+^ (+)	SiaD	*Pseudomonas aeruginosa*	Yes	Yes	(26)
Mg^2+^ (+)	DgcR	*Leptospira biflexa*	Yes	Yes	(27)
Mg^2+^ (+)	WspR	*Pseudomonas aeruginosa*	Yes^*b*^	Yes	(31)
Mg^2+^ (+)	Vc0395	*Vibrio cholerae*	Yes	Yes	(32)
Mg^2+^ (+)	PleD	*Caulobacter crescentus*	Yes	Yes	(25, 28)
Mg^2+^ (+)	tDGC	*Thermotoga maritima*	Yes	No	(29)
Mg^2+^ (+)	XAC0610	*Xanthomonas citri*	No	Yes	(33)
Mg^2+^ (+)	DgcB	*Bdellovibrio bacteriovorus*	Yes^*b*^	Yes	(34)
Mg^2+^ (+)	DosC	*Escherichia coli*	Yes	Yes	(35)
Mg^2+^ (+)	CD1420	*Clostridium difficile*	No	Yes	(36)
Mg^2+^ (+), **Zn^2+^ (−)-CZB domain**	DgcZ	*Escherichia coli*	Yes	Yes	(37)
Mg^2+^ (+), **Fe^2+^ (+)-GCS domain**	HemDGC	*Desulfotalea psychrophila*	No	Yes	(38)
Mg^2+^ (+), **Fe^2+^ (+)-Partner protein IsmP**	ImcA	*Pseudomonas aeruginosa*	Yes	Yes	(39)
Mg^2+^ (+), **Fe^2+^ (+)-GCS domain**	BpeGReg	*Bordetella pertussis*	No	Yes	(40)
Mg^2+^ (+), **Fe^2+^ (+)-GCS domain**	PcGCS	*Pectobacterium carotovorum*	No	Yes	(41)
Mg^2+^ (+)	DgcA	*Rhodobacter sphaeroides*	No	Yes	(30)
Mg^2+^ (+)	Slr1143	*Synechocystis sp. PCC6803*	No	Yes	(30)
Mg^2+^ (+), Ca^2+^ (−), Cu^2+^ (−), Zn^2+^ (−), and Ba^2+^ (−), Fe^2+^ and Co^2+^ (partial activity)	DgcA (SII1687)	*Synechocystis sp. PCC6803*	No	Yes	(42)

^*a*^ The table summarizes metal ions involved in regulating their GGDEF domains. Metal ions binding to N-terminal sensory domains are indicated in bold. CZB—chemoreceptor zinc binding; GCS—globin-coupled sensor.

^*b*^ Crystal structure did not have bound Mg^2+^ ion in the active site. (+) indicates ions promoting the activity of DGC; (−) indicates ions inhibiting the activity of DGC.

Like GGDEFs, Mg²^+^ and Mn²^+^ are commonly associated with catalytic activation in EAL proteins (Fig. 1A) (Table 2) (7, 43–45). The catalytic function of these PDEs is dependent on the EAL motif and the conserved DDFG(T/A)GYSS sequence within the EAL loop coordinating metal binding. Two metals are bound by EALs, both of which interact with the conserved EAL and DDFGTGYSS motif (46). The metal-bridging water is hypothesized to generate a hydroxide ion that performs nucleophilic attack on c-di-GMP, producing pGpG. The catalytic efficiency correlates with the Lewis acidity of the metal ions, with Mn²^+^ being more effective than Mg^2+^ (35). Crystal structures of EAL domains also reveal two conserved metal-binding sites typically coordinated by Mg²^+^ or Ca^2+^. However, structures of PA3825 from *P. aeruginosa* and CC3396 from *Caulobacter crescentus* in complex with the reaction product pGpG revealed a third, distinct metal-binding site (47). While the first two metal ions are required for nucleophile activation and phosphodiester bond cleavage, the third metal is positioned too far from the nucleophile to participate directly in catalysis (47). The third metal is observed specifically in the pGpG product-bound state, thereby stabilizing the negatively charged divalent state and product during hydrolysis (47).

**TABLE 2:** Metal ion regulation of EAL domain containing PDEs^*a*^

Metals regulating EAL domain containing PDEs	Protein	Organism	Crystal structure	*In vitro* protein activity tested	Reference
Ca^2+^ (−)	YkuI	*Bacillus subtilis*	Yes	Yes	(48)
Mn^2^ (+), Ca^2+^ (−)	PA3825	*Pseudomonas aeruginosa*	Yes	Yes	(47)
Mg^2+^ (+), Ca^2+^ (−)	Vc0395	*Vibrio cholerae*	Yes	Yes	(49)
Mg^2+^ (+), Mn^2+^ (+), Ca^2+^ (−)	YahA	*Escherichia coli*	Yes	Yes	(45, 50)
Ca^2+^ (−)	Bd1971	*Bdellovibrio bacteriovorus*	Yes	Yes	(51)
Mn^2+^ (+)	BlrP1	*Klebsiella pneumoniae*	Yes	Yes	(52, 53)
Mg^2+^ (+), Ca^2+^ (−) **Fe^2+^ (+)-PAS domain**	DosP	*Escherichia coli*	Yes	Yes	(54)
Mg^2+^ (+), Ca^2+^ (−)	CC3396	*Caulobacter crescentus*	No	Yes	(44)
Mg^2+^ (+)	RocR	*Pseudomonas aeruginosa*	No	Yes	(55)
Mg^2+^ (+), Mn^2+^ (+), Ca^2+^ (−), Zn^2+^ (−)	VieA	*Vibrio cholerae*	No	Yes	(56)
Mg^2+^ (+)	DcpA	*Mycobacterium smegmatis*	Yes	Yes	(57)
Mg^2+^ (+)	CD0757	*Clostridium difficile*	No	Yes	(36)
Mg^2+^(+), Mn^2+^(+), Ca^2+^(−), Zn^2+^ (−), Fe^2+^ (−)	ZpdA	*Vibrio cholerae*	No	Yes	(58)
Mn^2+^ (+), **Fe^2+^ (+**)	AxpdeA1	*Komagataeibacter xylinus*	No	Yes	(59)
Mg^2+^ (+), **Fe^2+^ (−)-NosP domain**	CdpA	*Vibrio cholerae*	No	Yes	(60)
Mn^2+^ (+)	FimX	*Pseudomonas aeruginosa*	Yes	Yes	(61)
Mg^2+^ (+)	MtbPDE	*Mycobacterium tuberculosis*	No	Yes	(62)
Mn^2+^ (+), Co^2+^ (+), Mg^2+^, Ni^2+^ (partial activity)	TBD1265	*Thiobacillus denitrificans*	Yes	Yes	(43)
Mn^2+^ (+)	TBD1269	*Thiobacillus denitrificans*	No	Yes	(43)
Mn^2+^ (+)	TBD1660	*Thiobacillus denitrificans*	No	Yes	(43)
Mn^2+^ (+)	CV0542	*Chromobacterium violaceum*	No	Yes	(43)
Mn^2+^ (+)	CV2505	*Chromobacterium violaceum*	No	Yes	(43)
Mn^2+^ (+)	NE0566	*Nitrosomonas europea*	No	Yes	(43)
Mn^2+^ (+), Mg^2+^, Co^2+^ (partial activity)	SFV_3559	*Shigella flexneri*	No	Yes	(43)
Mn^2+^ (+)	SO2039	*Shewenella oneidensis*	No	Yes	(43)
Mg^2+^ (+), Ca^2+^ (−)	PDE-A	*Komagataeibacter xylinus*	No	Yes	(21)

^*a*^ The table metal ions involved in regulating their EAL domains and additional cofactors bound to N-terminal sensory domains when known. (+) indicates ions promoting the activity of EAL; (−) indicates ions inhibiting the activity of EAL. Metals binding to N-terminal domain are bolded.

Dual-domain GGDEF and EAL enzymes are also common. When tested independently, the DGC and PDE domains of dual-domain proteins MorA, PA0575 (RmcA), CD0522, RbdA, BphG1, MSDGC1, and MtbDGC are active in the presence of Mg²^+^ (Table 3). The role of other metals like Mn^2+^ in controlling the catalytic activity of these dual-domain proteins has not been studied.

**TABLE 3:** Metal ion regulation of dual-domain GGDEF-EAL containing proteins^*a*^

Metals regulating DGC domain	Metals regulating EAL domain	Protein	Organism	Crystal structure	*In vitro* activity tested	Reference
Mg^2+^ (+)	Mg^2+^ (+)	MorA	*Pseudomonas aeruginosa*	Yes	Yes	(63)
Mg^2+^(+), Fe^2+^ (+)	Mg^2+^(+), Fe^2+^ (−)	CdgA	*Streptomyces ghanaensis*	No	Yes	(64)
Ca^2+^ (−)	Mg^2+^ (+)	PA0575	*Pseudomonas aeruginosa*	Yes	Yes	(65)
Mg^2+^ (+)	Mg^2+^ (+)	CD0522	*Clostridium difficile*	No	Yes	(36)
Mg^2+^ (+)	Mg^2+^ (+)	RbdA	*Pseudomonas aeruginosa*	Yes	Yes	(66, 67)
Mg^2+^ (+)	Mg^2+^ (+)	BphG1	*Rhodobacter sphaeroides*	No	Yes	(68)
Mg^2+^ (+)	Mg^2+^ (+)	MSDGC1	*Mycobacterium smegmatis*	No	Yes	(69)
Mg^2+^ (+)	Mg^2+^ (+)	MtbDGC	*Mycobacterium tuberculosis*	No	Yes	(62)

^*a*^ The table summarizes metal ions involved in regulating GGDEF and EAL domains. (+) indicates ions promoting the activity of the respective domain; (−) indicates ions inhibiting the activity of respective domains.

## HD-GYP CONTAINING PROTEINS REQUIRE FE²⁺, MN²⁺, OR MG²⁺ FOR ACTIVITY

Unlike GGDEF and EAL domains, the role of divalent metals in HD-GYP PDE activity is more complex, as these enzymes can also utilize Fe^2+^ to mediate PDE activity. HD-GYP PDEs belong to the HD domain superfamily of metal-dependent phosphohydrolases (70). These enzymes hydrolyze c-di-GMP into linear pGpG or GMP, with catalysis dependent on conserved motifs: the tandem histidine-aspartate (HD) sequence and the GYP residues in the loop located near the active site. HD-GYP domains typically coordinate two divalent metal ions, and mutational analyses of metal-binding residues have demonstrated their essential role in enzymatic activity. Although most characterized HD-GYPs utilize Fe²⁺, Mn²⁺, or Mg²⁺ for activity, other metals can influence the function of HD-GYPs (71) (Fig. 1A) (Table 4). In *V. cholerae*, a systematic analysis of all HD-GYP proteins found that only four are active *in vitro* and *in vivo*; however, these assays were performed exclusively with Mg²⁺, leaving the potential roles of other metals unresolved (72). Although most of the HD-GYPs bind two metal ions, PmGH from *Persephonella marina* EX-H1 binds three Fe²⁺ ions near the catalytic center, and mutation of metal-binding residues significantly impairs activity, underscoring the importance of metal coordination (73). Similarly, TM0186, from *Thermotoga maritima*, can bind three metal ions, and Fe^2+^, Fe^3+^, and Fe–Mn can confer PDE activity. While the binding of two metal ions enables cleavage of c-di-GMP to pGpG, the binding of a third Fe^2+^ facilitates the hydrolysis of pGpG to two GMPs (74). Moreover, crystal structures of the cyclic di-AMP PDEs, DhhP and PgpH, related to HD-GYP enzymes reveal Fe–Mn and Fe–Fe metal centers that may also be conserved in other HD-GYP PDEs (75, 76). Metal specificity of HD-GYPs may reflect environmental adaptation. Under anaerobic conditions, the intracellular levels of free Fe^2+^ increase 3–10 fold, and the manganese levels decrease more than 30-fold (77, 78). Hence, the metal centers may change depending on the environment, with Fe²⁺-dependent HD-GYPs favored under anaerobic conditions, while Mn²⁺-dependent enzymes may be favored in aerobic environments. Overall, HD-GYP proteins appear to utilize distinct metal cofactors in response to environmental cues, though only a few of the metals directly regulating the HD-GYP domain have been characterized to date.

**TABLE 4:** Metal ions regulation of HD-GYP domain-containing EALs^*a*^

Metals regulating HD-GYP domain containing EALs	Protein	Organism	Crystal structure	*In vitro* activity tested	Reference
Fe^2+^ (+), Co^2+^ (+)	SO3491	*Shewenella oneidensis*	No	Yes	(71)
Fe^2+^ (+)	VCA0681	*Vibrio cholerae*	No	Yes	(71)
Fe^2+^ (+), Co^2+^ (+)	PmGH	*Persephonella marina EX-H1*	Yes	Yes	(73)
Mn^2+^ (+), Mg^2+^ (+)	PA4108	*Pseudomonas aeruginosa*	No	Yes	(79)
Fe^2+^ (ND)	Bd1871	*Bdellovibrio bacteriovorus*	Yes	No	(80)
Mg^2+^ (+)	VC1295	*Vibrio cholerae*	No	Yes	(72)
Mg^2+^ (+)	VCA0210	*Vibrio cholerae*	No	Yes	(72)
Mg^2+^ (+)	VCA0681	*Vibrio cholerae*	No	Yes	(72)
Mn^2+^ (+), Fe^2+^ (−), Mg^2+^ (−), Cu^2+^ (−), Co^2+^ (−), Ni^2+^ (−), Zn^2+^ (−), **Fe^2+^+ O_2_ (+), Fe^3+^ (+**)	AL536_01530	*Vibrio fluvialis*	No	Yes	(81)

^*a*^ The table summarizes metal ions involved in regulating their HD-GYP domain and additional cofactors bound to N-terminal sensory domains when known. (+) indicates ions promoting the activity of HD-GYP; (−) indicates ions inhibiting the activity of HD-GYP. Metals binding to N-terminal domain are bolded.

## IMPACT OF MIS-METALATION ON CATALYSIS OF C-DI-GMP CATABOLIZING PROTEINS

The bacterial cytoplasm has a diverse range of metals at different concentrations. In wild-type *E. coli*, intracellular concentrations of metal ions vary widely, with Mg being the most abundant at 10^8^ atoms per cell (>10 mM), Ca, Zn, and Fe less abundant at 10^5^ atoms per cell (0.1 mM), and Mn being the least abundant metal described in this review at 10^4^ atoms per cell (∼10 μM) (82). It is important to note that many of these metals are complexed with other macromolecules, so these values do not represent the concentration of free metals in the cytoplasm. Therefore, it is not surprising that apart from Mg^2+^ and Mn,^2+^ other metals can bind to the catalytic site of GGDEFS, EALs, and HD-GYPs to modulate the activity of these enzymes.

The presence of Ca²^+^ in crystal structures often correlates with the loss of enzymatic activity in EAL proteins (7, 43–45). Apart from Ca^2+^, Zn^2+^ and Fe^2+^ inhibit the PDE activity of EAL proteins (7, 58) (Fig. 1A) (Table 2). Binding of Zn²^+^ and Ca²^+^ to the metal-binding center potentially perturbs the structure and formation of hydroxide ion, thereby inhibiting the PDE activity (52). This mis-metalation phenomenon governed by the Irving-Williams series and environmental metal availability has been observed under various metal stress conditions for many enzymes, where stronger binding metal ions, such as Zn^2+^ and Ca^2+^, replace Mg^2+^ and Mn^2+^ (18, 83). However, the EAL domain TBD1265 was significantly activated by Ni²^+^ and Co²^+^ *in vitro* at 0.5 mM Ni²^+^ and Co²^+^, highlighting the potential diversity of metal-dependent regulation of EALs (43). Whether Ni²^+^ and Co²^+^ activate EAL enzymes *in vivo* at more physiologically relevant concentrations and specific environments at which this may happen remains to be explored.

Some HD-GYPs also demonstrate selective metal binding and activity. The HD-GYP SO3491 from *Shewanella oneidensis* and VCA0681 from *V. cholerae* binds Fe²^+^, Co²^+^, Ni²^+^, and Mn²^+^, yet it is active exclusively with Fe^²+^ (71). Therefore, while the role of Mg^2+^, Mn^2+^, and Fe^2+^ in promoting activity of c-di-GMP catabolic enzymes is clear, the impact of other divalent metals on catalysis is more varied and less understood.

## METALS FUNCTIONING AS SIGNALS TO IMPACT THE ACTIVITY OF C-DI-GMP METABOLIZING ENZYMES

Apart from directly impacting the catalytic activity of GGDEF, EAL, and HD-GYP-encoding enzymes, divalent metals can regulate the activity of these enzymes by interacting with the associated N-terminal signaling domains or modulating protein–protein interactions (Fig. 1B). In *E. coli*, Zn²^+^ inhibits the DgcZ DGC by binding to its N-terminal CZB domain, locking the enzyme into a misaligned homodimer incapable of binding to two GTPs and consequently suppressing c-di-GMP synthesis and biofilm formation (37). An example of Fe^3+^ increasing c-di-GMP synthesis of a DGC was reported in *P. aeruginosa*. High iron levels result in Fe^3+^ binding to CHASE4 domain of IsmP DGC. This binding triggers a conformational change that relieves IsmP inhibition of partner protein ImcA, thereby promoting DGC activity of ImcA (39). In *V. fischeri*, Ca²^+^ acts as a signal via the DGC CasA by binding to N-terminal dCache-1 domain, thereby enhancing biofilm formation and delaying dispersal (84, 85). Though HD-GYPs encode diverse N-terminal sensory domains, whether metals bind to these domains and regulate the activity of HD-GYPs has not been demonstrated. Some of the HD-GYPs contain an additional metal-binding HD domain in the N-terminal region, apart from its C-terminal HD-GYP domain, but mutation of the metal-binding residues in the N-terminal HD domain of VCA0681 and SO3491 did not influence the C-terminal activity of the HD-GYP domain (71). In *Vibrio fluvialis*, the HD-GYP protein encoded by AL536_01530 contains a globin-coupled sensor N-terminal domain and a C-terminal HD-GYP domain. The PDE activity of AL536_01530 is induced by the binding of Fe^3+^ or Fe^2+^ in the presence of oxygen, along with Mn^2+^ (81). The Fe^3+^ or Fe^2+^ with oxygen binds the N-terminal globin-coupled sensor domain, and Mn^2+^ binds to the C-terminal HD-GYP domain, modulating PDE activity (81).

## DIVALENT METALS THAT MODULATE C-DI-GMP LEVELS VIA UNKNOWN MECHANISM

In some cases, metal addition alters cellular c-di-GMP concentrations, but it is yet unclear if the metal functions during catalysis or as a signal (Fig. 1C). For example, in line with the *in vitro* activation of PDEs by Mn²^+^, in *P. aeruginosa*, extracellular Mn²^+^ stimulates dispersal by activating the PDE RbdA (86). In *V. cholerae*, Zn^2+^ directly inhibits the activity of the EAL-containing PDE ZpdA at low micromolar levels, overcoming the positive effect of Mg^2+^ and Mn^2+^ (58). This increases c-di-GMP concentrations, driving *V. cholerae* to a biofilm state. As Zn^2+^ concentrations are elevated in chitin, it was hypothesized that the role of zinc in this system could be to drive biofilm formation on environmental chitin surfaces (58). For both RbdA and ZpdA, whether such metal regulation is by interaction at the active site or via a signaling domain has not been resolved. In *Bordetella bronchiseptica*, Ca²^+^ in the presence of bovine serum albumin represses biofilm formation by activating *in vivo* EAL activity, in contrast to Ca^2+^ inactivating EAL domain activity *in vitro* (87). Whether the *in vivo* Ca^2+^ effect was due to direct modulation of EAL activity or indirect effects remains to be determined. Divalent metals were shown to affect c-di-GMP related phenotypes, such as motility, flagellation, and biofilm formation, in other bacterial species (88–91); however, whether they impact c-di-GMP-dependent enzymes directly is not understood.

## METALS IMPACTING C-DI-GMP SIGNALING THROUGH TRANSCRIPTIONAL CONTROL

Divalent metals shape bacterial physiology not only through directly modulating enzymatic activity but also via metalloregulatory transcription factors. Chief among these are members of the FUR family, including Fur (+Fe^2+^) and Zur (+Zn^2+^), which sense intracellular metal levels and modulate gene expression to import or export cognate metals to maintain metal homeostasis (reviewed extensively in (92–94). In *V. cholerae*, Fur activates expression of the *vieS* PDE and represses the DGCs *cdgD* and *vca0560*, resulting in decreased c-di-GMP levels (Fig. 1D) (95, 96). Similarly, in *Yersinia pestis*, Fur reduces c-di-GMP levels and biofilm formation by repressing the DGC *hmsT* (97) (Fig. 1D). In contrast to Fur, in *V. cholerae*, Zur with Zn²^+^ inhibits the transcriptional activity of PDE ZpdA, thereby increasing intracellular c-di-GMP levels (Fig. 1D). In line with this result, a *zur* mutant of *V. cholerae* has decreased expression of biofilm-related genes (58, 98). Although biofilm-related genes have been shown to be regulated by Fur and Zur in several other bacteria (97, 99–102), whether this regulation is mediated by c-di-GMP-mediated genes remains to be explored.

## CONCLUSIONS AND FUTURE PERSPECTIVES

Despite extensive *in vitro* studies, the physiological relevance of metal-dependent regulation of c-di-GMP remains underexplored. Many structural studies of DGCs and PDEs rely on crystallization conditions rich in Mg²^+^, potentially obscuring the roles of other biologically relevant metals. Moreover, c-di-GMP-related signaling and regulation were mostly studied in rich media that contain elevated levels of divalent metals, which may not reflect environmental conditions (19). For example, Zn²^+^ and Ca²^+^ are high in chitin-rich environments and seawater (58, 103), where bacteria like *Vibrio* may experience inhibition of specific EAL proteins, promoting biofilm formation. In the human host, metal starvation and intoxication are strategies used to combat pathogenic bacteria (reviewed in 13, 20). Similarly, microbes colonizing plants experience both metal starvation and toxicity that influence colonization and community dynamics (104). Which DGCs and PDEs are activated or repressed under different metal stress conditions and how these changes influence c-di-GMP levels and physiological outcomes remain to be determined.

Many c-di-GMP-metabolizing enzymes and metal transporters are membrane-bound, suggesting potential crosstalk between metal uptake and local c-di-GMP signaling. Localized concentrations of c-di-GMP can elicit specific cellular responses, while global levels govern broader physiological changes (8, 105–107). Similarly, localized metal concentrations may selectively activate or inhibit specific DGCs or PDEs. Metal analysis of single-celled *Chlamydomonas reinhardtii* using X-ray fluorescence microscopy revealed distinct metal distribution in different organelles (108), and specific metals are enriched in different regions of the bacterial cells. For example, the cell envelope is enriched in Mg and Ca; membranes are enriched in Fe, Ca, Mn, and Mg; soluble proteins are enriched in Zn, Fe, Mn, and Mg; and nucleic acids are enriched in K and Mg (reviewed in 18). DGCs and PDEs that possess transmembrane domains could be activated by Mg or Mn present in the microenvironment of membranes upon membrane disruption. In addition to directly binding to and modulating the activity of DGCs and PDEs, metal concentrations can impact protein-protein interactions that impact signaling. For example, in *V. cholerae*, the PDE activity of MbaA depends on its partner NspS, which senses spermidine and norspermidine (106). Similarly, in *Agrobacterium tumefaciens*, the dual-domain DGC–PDE protein DcpA is controlled by the pterin-binding protein PruR (109). Because many solute-binding proteins can bind metals (110), the fluctuating concentrations of metals in the cell could modulate the interaction of these solute-binding proteins, impacting c-di-GMP signaling through the associated DGC or PDE. Advanced imaging techniques, such as synchrotron-based single-cell X-ray fluorescence microscopy (XFM), could illuminate the subcellular distribution of metals and c-di-GMP, offering insights into their spatial regulation. Like metals, c-di-GMP levels are also maintained at homeostatic levels to efficiently switch between motility and sessility, as high levels of c-di-GMP can hinder survival and colonization (111–114). For example, Ca^2+^ influences both biofilm formation and dispersal in *V. fischeri*, while Mn^2+^ affects initial attachment and dispersal at later stages in *P. aeruginosa* (84, 86). These dynamics highlight the importance of time-course studies to capture the transient nature of metal and c-di-GMP interactions.

Overall, metal regulation of PDEs and DGCs is highly species-specific, highlighting the nuanced and context-dependent roles of extracellular metals in shaping intracellular c-di-GMP signaling and associated phenotypes. Importantly, most studies have not quantified intracellular metal concentrations, which may vary across species despite tight homeostatic control and could significantly influence observed phenotypic outcomes. The same metal ion can exert opposing effects depending on the bacterial species, the specific regulatory enzymes involved, and the environmental conditions, emphasizing the need to consider the ecological context of bacterial colonization when interpreting metal–c-di-GMP interactions. Future research should, therefore, aim to systematically dissect intracellular metal dynamics, comprehensively map metalloregulator regulons, and define their direct impact on DGC and PDE activities. Investigating these relationships in diverse environments will be crucial for understanding microbial behavior and developing novel strategies to control biofilm-related infections. Metal-dependent regulation of c-di-GMP signaling is a multifaceted, dynamic, and environmentally responsive process. A deeper understanding of metal-c-di-GMP interplay across spatial, temporal, and ecological scales will illuminate new dimensions of bacterial signaling and community behavior.
